# IFNG +874T/A polymorphism is not associated with American tegumentary leishmaniasis susceptibility but can influence *Leishmania *induced IFN-γ production

**DOI:** 10.1186/1471-2334-7-33

**Published:** 2007-04-24

**Authors:** Guilherme Inocêncio Matos, Claudia de J Fernandes Covas, Rita de Cássia Bittar, Adriano Gomes-Silva, Fabiana Marques, Viviane C Maniero, Valdir S Amato, Manoel P Oliveira-Neto, Marise da Silva Mattos, Claude Pirmez, Elizabeth P Sampaio, Milton O Moraes, Alda Maria Da-Cruz

**Affiliations:** 1 Laboratório de Imunoparasitologia, Departamento de Imunologia, Instituto Oswaldo Cruz, FIOCRUZ, Rio de Janeiro, Brazil; 2 Laboratório de Hanseníase, Departamento de Micobacterioses, Instituto Oswaldo Cruz, FIOCRUZ, Rio de Janeiro, Brazil; 3 Laboratório de Imunopatologia, Departamento de Bioquímica e Biologia Molecular, Instituto Oswaldo Cruz, FIOCRUZ, Rio de Janeiro, Brazil; 4 Ambulatório de Leishmanioses, Divisão de Clínica de Moléstias Infecciosas e Parasitárias, Faculdade de Medicina, Universidade de São Paulo, São Paulo, Brazil; 5 Instituto de Pesquisa Clínica Evandro Chagas (IPEC), FIOCRUZ, Rio de Janeiro, Brazil

## Abstract

**Background:**

Interferon-gamma is a key cytokine in the protective responses against intracellular pathogens. A single nucleotide polymorphism (SNP) located in the first intron of the human IFN-γ gene can putatively influence the secretion of cytokine with an impact on infection outcome as demonstrated for tuberculosis and other complex diseases. Our aim was to investigate the putative association of IFNG+874T/A SNP with American tegumentary leishmaniasis (ATL) and also the influence of this SNP in the secretion of IFN-γ *in vitro*.

**Methods:**

Brazilian ATL patients (78 cutaneous, CL, and 58 mucosal leishmaniasis, ML) and 609 healthy volunteers were evaluated. The genotype of +874 region in the IFN-γ gene was carried out by Amplification Refractory Mutational System (ARMS-PCR). *Leishmania*-induced IFN-γ production on peripheral blood mononuclear cell (PBMC) culture supernatants was assessed by ELISA.

**Results:**

There are no differences between +874T/A SNP frequency in cases and controls or in ML versus CL patients. Cutaneous leishmaniasis cases exhibiting AA genotype produced lower levels of IFN-γ than TA/TT genotypes. In mucosal cases, high and low IFN-γ producers were clearly demonstrated but no differences in the cytokine production was observed among the IFNG +874T or A carriers.

**Conclusion:**

Our results suggest that +874T/A polymorphism was not associated with either susceptibility or severity to leishmaniasis. Despite this, IFNG +874T/A SNP could be involved in the pathogenesis of leishmaniasis by influencing the amount of cytokine released by CL patients, although it could not prevent disease development. On the other hand, it is possible that in ML cases, other potential polymorphic regulatory genes such as TNF-α and IL-10 are also involved thus interfering with IFN-γ secretion.

## Background

American tegumentary leishmaniasis (ATL) is a vector born disease caused in Brazil mainly by *Leishmania (Viannia) braziliensis*, an intracellular pathogen. This unique *Leishmania *species can cause a spectrum of clinical presentation ranging from self-healing or benign cutaneous lesions (CL) to more severe forms, such as mucosal leishmaniasis (ML). ML is not as common as CL, affecting approximately 5% of patients infected almost exclusively by *L. braziliensis *[1], indicating that host intrinsic factors can predispose for severity. The outcome of infection is profoundly influenced by the balance between effector and regulatory specific T-cell responses in which a higher immunoresponsiveness to leishmanial antigens accounts for worsened prognosis of the disease [2-5].

Susceptibility to infectious diseases is influenced by the genetic background and it is supposed that efficient activation of cellular immune response specifically the IFNG/IL-12/23 axis might play a key role in protection [6]. In leishmaniasis, cytokines such as interferon-gamma (IFN-γ) and tumor necrosis factor alpha (TNF-α) have a major role in controlling intracellular growth of the etiological pathogen. Suitable amounts of TNF-α and IFN-γ produced by subclinical or CL patients are proven to be important in mediating cure, but over expression of both cytokines generated in consequence of exacerbated immune response can induce intense pathological damage, as observed in ML [3,4,7-9]. Importantly, clinical cure of ML is directly related to a decrease in TNF-α levels [8-10], but not IFN-γ [4]. Despite this, even long-term cured ML patients still produce higher IFN-γ levels than CL indicating that some individuals maintain their ability to continuously produce significant amounts of this cytokine [4]. Interestingly, high and low IFN-γ producers are clearly seen among cured ML patients [4]. Such data suggest that genetic factors potentially drive IFN-γ secretion upon leishmanial stimuli and have the potential to determine the clinical evolution or even predisposition for more severe forms of the disease.

A single nucleotide polymorphism (SNP) located in the first intron of the human IFN-γ gene, at the 5' end adjacent to a CA repeat region (+874T/A polymorphism) can putatively influence the secretion of IFN-γ [11]. Analysis of the biological role of this SNP suggested that +874A carriers are low IFN-γ producers [12]. This finding can explain why a high frequency of +874A allele has been eventually associated with susceptibility to tuberculosis in different population [12-16]. However, this SNP is a still controversial issue regarding risk for acquiring tuberculosis since no association with susceptibility to this disease was demonstrated by other authors [17] even when larger and ethnically different population were studied [18]. Susceptibility to other infectious diseases like severe acute respiratory syndrome (SARS) have also been described [19] suggesting that variability in IFN-γ production linked to this SNP is possibly playing a major role in susceptibility to infectious diseases, especially intracellular pathogens.

Previous reports have shown that polymorphism located in the TNF-α promoter -308A were in a higher frequency among ML patients [20], but no data is available for the IFN-γ genotypic analysis in ATL. It is conceivable that allelic variants in IFN-γ genes may influence the levels of released protein [11] which in turn could increase the susceptibility to leishmaniasis or predispose for progression to mucosal leishmaniasis. Our aim was to investigate whether +874T/A of IFN-γ gene increase the risk for developing cutaneous or mucosal leishmaniasis as well as to determine whether high and low *Leishmania*-induced IFN-γ production can be associated with the existence of this polymorphism.

## Methods

### Patients

A total of 136 leishmaniasis patients were enrolled in this study. Seventy-eight CL patients (50 males and 28 females; mean age ± SD = 34 ± 16 years) and 58 ML (39 males and 19 females; mean age ± SD = 58 ± 11 years) were included. Patients were referred from endemic areas of *L. braziliensis *infection in the state of Rio de Janeiro, Brazil. The diagnostic criteria were based on clinical, parasitological and immunological parameters as described elsewhere [4]. Patients were reexamined one year after the end of the therapy and were considered clinically cured at the moment of this study. A total of 609 healthy individuals (288 females and 321 males) from the same geographic area (Rio de Janeiro, Brazil) were involved in the study. Written informed consent was obtained from all individuals according to protocol approved by the Ethical Committee for Human Research of the Fundação Oswaldo Cruz, Brazilian Ministry of Health.

### Genotyping of +874T/A SNP

Genomic DNA was extracted using the commercial kit DNAzol (DNAzol Invitrogen Life Technologies, Gaithersburg, MD, USA) according to the manufacture's instructions. The genotyping of the +874 region in the first intron of the IFN-γ gene was carried out by Amplification Refractory Mutational System (ARMS-PCR), with a reaction for amplification for each allele (A or T) as described previously [11].

### Peripheral blood mononuclear cell culture and IFN-γ measurement

PBMC from a total of 60 patients (31 ML and 29 CL) was obtained by Ficoll-Hypaque density centrifugation and the cells (3 × 10^6 ^per well) stimulated *in vitro* for 5-days with crude extract of *L. braziliensis *promastigote antigens (50 μg/well), as described previously [4], when supernatants were recovered and measured by ELISA. The monoclonal antibodies and recombinant cytokines were purchased from Pharmingen (San Diego, CA, USA). The procedures were performed according to the manufacture's instructions. Samples were tested in duplicate and the concentration was analyzed using the SOFTmax^®^PRO 4.0 program (Life Sciences Edition, Molecular Devices Corporation, USA). Results were expressed in picograms per milliliters (pg/mL). The minimum IFN-γ levels detected were 62.5 pg/mL.

### Statistical analysis

The case-control study was analyzed using Logistic Regression Model with correction for sex as a covariate, performed in statistical software, The R Foundation for Statistical Computing, version 2.1.1 [21].

Results obtained from quantitative variables were analyzed by One-way Analysis of Variance (ANOVA) with Tukey-Kramer post-test, using GraphPad Prism version 3.00 for Windows (GraphPad Software, San Diego, CA, USA).

## Results

The genotypic and allelic frequencies of healthy controls as well as the groups of patients divided in the clinical forms of ATL are shown in Table 1. Both populations were found under Hardy-Weinberg Equilibrium for this *locus*. Analyses using the genotypic and allelic frequencies when comparing patients and controls demonstrated no association with susceptibility or protection to ATL *per se *(p = 0.59 for genotypes and p = 0.4735 for alleles). In the same way, no associations with the severe mucosal form were found when compared to CL cases (p = 0.561). Despite the lower frequency of TT genotype among ATL in comparison to AA or TA patients, no statistical difference was observed in comparison to controls or among the patient population. Indeed the low frequency of TT genotype is comparable to the one already expected in the general population (control individuals).

**Table 1:** Distribution of genotypic and allelic frequencies among American tegumentary leishmaniasis (ATL) patients (mucosal and cutaneous forms) and healthy controls.

Subjects studied	IFN-γ (+874 T/A) genotypes number of cases (%)			Allele frequencies (%)	
		
	AA	TA	TT	A	T
ATL patients	57 (0.42)	62 (0.46)	17 (0.12)	0.59	0.41
Cutaneous leishmaniasis	30 (0.38)	38 (0.49)	10 (0.13)	0.63	0.37
Mucosal leishmaniasis	27 (0.47)	24 (0.41)	7 (0.12)	0.67	0.33
Healthy controls	224 (0.37)	273 (0.45)	112 (0.18)	0.59	0.41

**Figure 1:**
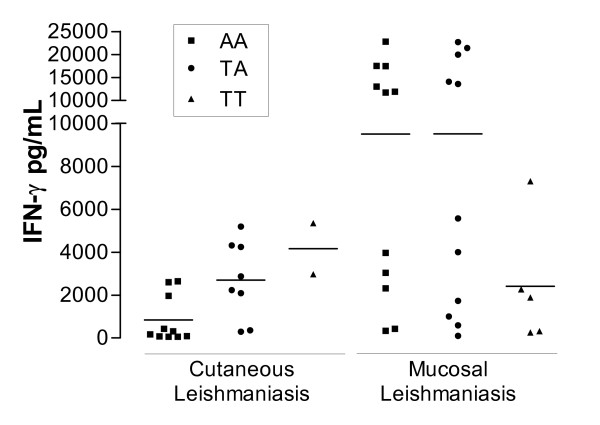
IFN-γ levels detected in *Leishmania *antigens stimulated peripheral blood mononuclear cell culture supernatants from patients with cutaneous and mucosal leishmaniasis divided according the genotype group. Each symbol represents one patient. Symbols refer to ■ AA, ● TA and ▲ TT genotypes, respectively. Bars represent the median values.

The analysis of IFN-γ production upon *Leishmania *antigens stimulation showed, as expected, that differences in the IFN-γ secretion between CL and ML were very significant (p < 0.001). In order to evaluate the functional influence of SNP +874 in the production of IFN-γ (mean ± SD) observed in these clinical forms of ATL, a comparison of leishmanial induced cytokine levels produced in each genotype was carried-out (Figure 1). In ML patients, similar levels of IFN-γ were produced independently of the IFNG +874 genotype (p = 0.09): AA = 9,504 ± 7,858 pg/mL (median = 11,740 pg/mL; n = 11), TA = 9,522 ± 8,995 pg/mL (median = 5,582 pg/mL; n = 11) and TT = 2,406 ± 2,883 pg/mL (median = 1,884 pg/mL, n = 5). In addition, ML patients can be clearly (p < 0.0001) separated into two groups according to the levels of IFN-γ production: high (>10.000 pg/ml; 15,700 ± 5,866 pg/ml, n = 12) and low (<10.000 pg/ml; 2,191 ± 2,192 pg/ml, n = 15). This profile of IFN-γ producers was observed indistinctively among AA and TA genotypes. On the other hand, CL patients presented significant differences when IFN-γ production was compared among patients from the three IFNG +874 genotypes (p = 0.01). The detectable IFN-γ values were lower for +874AA individuals (844 ± 1,099 pg/mL, median = 244 pg/mL, n = 10); moderate for +874TA (2,700 ± 1,818 pg/mL, median = 2,556 pg/mL, n = 08) and high in +874TT (4,162 ± 1,678 pg/mL, n = 2). Pos-test indicates that significant differences were observed between +874AA and +874TA (p < 0.05). In 11 CL and 2 ML the IFN-γ values were under the minimum detectable levels.

## Discussion

A great deal of evidence indicates that susceptibility to leishmaniasis or other infectious diseases can be related to genetic variability at cytokine *loci *[15,20,22,23]. After exposition to *L. braziliensis*, a host can acquire infection without developing the disease, or progress through several stages from mild CL to severe ML disease. In *L. braziliensis *infection the downmodulation of a type 1 response predispose for the development of CL [7], while higher levels of IFN-γ are observed in association with mucosal disease [3,4].

Our results indicate that IFNG +874 SNP was not associated with development of leishmaniasis *per se *or progression to severe forms. However, a decreased frequency of the homozygous (TT) genotype was observed in CL and also in ML patients when compared to controls (Table 1). An association between reduced +874TT IFN-γ homozygous frequency and chronicity of the disease was also observed in Sicilian tuberculosis patients [16]. On the other hand, this SNP seems to impact on *in vitro *production of IFN-γ in CL, where the T allele was associated with higher levels of this cytokine. As expected for IFNG +874 polymorphism [11], cured CL cases exhibiting AA genotype presented significantly lower amounts of *Leishmania-*stimulated IFN-γ levels than TA/TT genotypes. Similar results were obtained in active tuberculosis patients and this profile is maintained even months after the clinical cure, indicating that the intrinsic patients' ability to produced IFN-γ was not affected [12]. Due to a still controversial role of this SNP in influencing the outcome of intracellular infections [16,17], it is possible that a larger or a different population (*eg *from other Brazilian endemic areas) would show a positive relationship with either severity or susceptibility to leishmaniasis as shown in tuberculosis patients.

In ML cases no differences in the IFN-γ production was observed among the IFNG +874 genotypes. It is possible that under an exacerbated response, which is observed in ML even after clinical cure [4], the presence (or absence) of the T allele does not affect the levels of IFN-γ. No association was observed between the time of cure after therapy and the levels of IFN-γ production. However, we can not rule out the hypothesis that longer period of illness may induce, in some patients, a sustained hyperresponsiveness to leishmanial antigens due to a chronic parasite activation [24]. Nevertheless, not only the presence of IFN-γ *per se *but also the secretion levels of others cytokines (like TNF-α and IL-10) constitute key factors in immunoregulating the host-parasite relationship [3,5,8,9], demonstrating that many genomic variations located in others candidate genes can contribute for phenotypic expression [25].

Taken together, our results reinforce the idea that IFNG +874 SNP has a functional role in the regulation of the gene in response to intracellular pathogens. However, it can be hypothesized that depending on the immunopathogenic characteristics influencing the clinical status of the patient (CL or ML) different signaling pathways are activated inducing distinct transcriptional factors [11,13] leading, as a consequence, to differential levels of IFN-γ production under similar stimulation conditions.

## Conclusion

Despite of being caused by the same *Leishmania *species, CL and ML patients have distinct immunoregulatory mechanisms induced by this parasite which can explain the difference on the pathogenesis, the therapeutic response and maybe the prognosis of the disease. The complex interactions which occur after parasite infection predict that the clinical course of the disease could not be explained by a unique mechanism. Our results demonstrate that IFNG +874T/A SNP could be involved in the pathogenesis of leishmaniasis by influencing the amount of IFN-γ released by CL patients, although it could not prevent disease development. It is possible that in ML cases other potential polymorphic regulatory genes such as TNF-α and IL-10 are also involved which in turn can influence the IFN-γ production.

## List of Abbreviations

Single nucleotide polymorphism – SNP

Interferon-gamma – IFN-γ

Tumor necrosis factor alpha – TNF-α

Interleukin 10 – IL-10

American tegumentary leishmaniasis – ATL

Cutaneous leishmaniasis – CL

Mucosal leishmaniasis – ML

Peripheral blood mononuclear cells – PBMC

Amplification refractory mutational system-Polymerase Chain Reaction – ARMS-PCR

Standard deviation – SD

## Competing interests

The authors declare that they have no competing interests. The authors do not have a commercial or other association that might pose a conflict of interest

## Authors' contributions

All authors read and approved the final manuscript. GIM, CJC, MOM, AMC contributed equally for study design, data collection, experimental procedures, interpretation of the results and manuscript elaboration. RCB, AGS, FM and VM participated on data collection and experimental procedures. VSA, MPON, MSM and AMC were responsible for clinical evaluation. CP and EPS participated on study design and helped on draft manuscript.

## Pre-publication history

The pre-publication history for this paper can be accessed here:


